# Chimeric autoantibody receptor T cells specifically eliminate Graves’ Disease autoreactive B cells

**DOI:** 10.3389/fimmu.2025.1562662

**Published:** 2025-04-08

**Authors:** Abigail Cheever, Hunter G. Lindsay, Chloe C. Kang, Mackenzie Hansen, Kimball Demars, Kim L. O’Neill, K. Scott Weber

**Affiliations:** Department of Microbiology and Molecular Biology, Brigham Young University, Provo, UT, United States

**Keywords:** CAR T cell, Graves’ disease, autoimmunity, autoantibody, autoantigen, CAAR T cell, B cell, T cell

## Abstract

**Introduction:**

Chimeric antigen receptor (CAR) T cells have recently become an important treatment for hematological cancers by efficiently eliminating B cells. B cell depleting CAR T cells are also in clinical trials for their use in treating severe autoimmune diseases and have shown promise in patients who have exhausted other treatment options; however, they do result in immunosuppression due to B cell depletion. Specifically eliminating the disease-causing B cells while leaving the healthy B cells untouched could address this limitation.

**Methods:**

A chimeric autoantibody receptor (CAAR) has an autoantigen as the binding domain of the CAR T cell and could allow for specific targeting of autoreactive B cell populations. In Graves’ Disease (GD), pathogenesis is centered around autoreactive B cells which are specific for thyroid stimulating hormone receptor (TSHR). By engineering epitopes of TSHR as the binding domain, our CAAR was able to bind to anti-TSHR antibodies and B cell receptors.

**Results:**

These TSHR CAAR T cells specifically eliminated anti-TSHR B cells, without exhibiting cytotoxicity against healthy B cells. We hypothesized that soluble autoantibodies and thyroid stimulating hormone (TSH) could bind to the CAAR, potentially causing overactivation or inhibition. When evaluated, we found that one construct was significantly impacted by soluble autoantibodies, while the other construct was uninhibited. Soluble TSH did not significantly affect either construct. The TSHR CAAR T cells were also effective at eliminating anti-TSHR B cells in the presence of plasma from various GD patients.

**Discussion:**

Thus, TSHR CAAR T cells show promise in eliminating the disease-causing autoreactive B cells in GD without eliminating healthy cells. This treatment mechanism also has the potential to be used in other B cell-mediated autoimmune diseases.

## 1 Introduction

Chimeric antigen receptor (CAR) T cell therapies have been successful in treating leukemias and lymphomas and there are currently seven FDA-approved therapies that target B cells using CD19 and B cell maturation antigen (BCMA) as targets (1, 2). Recently, B cell depleting CAR T cell therapies have also shown promise in treating severe B cell-mediated autoimmune diseases (3, 4). There are many ongoing clinical trials using anti-CD19 and anti-BCMA CAR T cells to treat autoimmune diseases including Systemic Lupus Erythematosus (SLE), Myasthenia Gravis, Systemic Sclerosis, Idiopathic Inflammatory Myopathy, and others (5, 6). Initial updates on these trials have been positive, lending credence to the idea that CAR T cells could be a viable therapy for autoimmune diseases (4). However, these B cell depleting CAR T cell therapies result in immunosuppression (3). While most patients recover B cell populations over time, there are instances where B cell populations do not return for months to years, and other instances where they never return due to CAR T cell persistence and memory responses against B cells (7).

In B cell-mediated autoimmune diseases with clearly established autoantigens, a novel CAR construct, called a chimeric autoantibody receptor (CAAR), can be used. CAAR T cells have an autoantigen as the binding domain which acts as bait for autoreactive B cells, interacting with the B cell receptors (BCRs) and allowing the CAAR T cell to selectively eliminate the autoreactive B cells (8). This technology was first developed for Pemphigus Vulgaris and has since been applied to Myasthenia Gravis as well (8, 9).

Graves’ Disease (GD) is an organ-specific, B cell-mediated autoimmune disease which affects the thyroid (10). GD pathogenesis is centered around autoreactive B cells, which produce anti-TSHR antibodies (Abs) that bind to thyroid stimulating hormone receptor (TSHR) on thyroid cells (11). These anti-TSHR Abs mimic thyroid stimulating hormone (TSH) and overstimulate TSHR, producing higher than necessary levels of thyroid hormone, including T3 and T4 (12, 13). The main manifestation of GD is hyperthyroidism; complications can occur where autoantibodies are cross-reactive with the eyes and heart, causing Graves’ Eye Disease and cardiomyopathy (14, 15). The current standard of treatment for GD starts with anti-thyroid drugs or radioiodine therapy to block thyroid function and, if these are not effective, a thyroidectomy is common practice (16). These treatments then require the patient to receive hormone replacement therapy for the duration of their life, as the thyroid is either no longer functional or present (17). Even after standard treatment, Graves’ Eye Disease and cardiomyopathy can still occur because autoreactive B cells and autoantibodies are still present (14, 18). A therapy that can target autoreactive B cells and the resulting autoantibodies could help to prevent or treat these complications of GD as well (19).

We have developed and engineered a CAAR T cell for GD designed to specifically eliminate the autoreactive B cells that cause GD. Multiple epitopes of TSHR were selected for the binding domain of the CAAR T cells, and then transduced into primary T cells for characterization. These CAAR T cells selectively eliminated anti-TSHR B cells with varying degrees of efficiency. CAAR T cell cytotoxicity and specificity was also examined when in the presence of soluble autoantibodies, TSH, and GD patient plasma.

## 2 Materials and methods

### 2.1 Plasmid design

#### 2.1.1 CAAR plasmid and TSHR epitope selection

CAAR plasmids were based on an anti-CD19 CAR plasmid (Addgene #135991, Watertown, MA). TSHR was modeled using AlphaFold 2 to predict which epitopes would fold properly (20). Selected epitopes were modeled individually, and their predicted structure was compared with the native protein. The cutoff points for each of the epitopes were refined to end up with epitopes that were predicted to bind as similarly to native TSHR as possible. Epitopes 1, 2, and 2.7 were then synthesized (TwistBiosciences, South San Fransisco, CA) with a GS linker attached to the C terminus and BpiI restriction sites at either end to facilitate cloning into the CAR plasmid. Following cloning, whole plasmid sequencing was performed (Plasmidsaurus, Monrovia, CA) to confirm proper cloning.

#### 2.1.2 Anti-TSHR BCR plasmid

A B cell co-receptor plasmid was designed with CD79a and CD79b to facilitate the expression of the anti-TSHR BCRs (**Supplementary Figure S1A**). This plasmid was constructed by VectorBuilder (Chicago, IL). To create patient-based anti-TSHR B cell lines, we used sequences for anti-TSHR Abs that were derived from GD patients (M22 and K1-18) (21). The heavy and light chain variable regions of this sequence were combined with an IgG1 constant region and transmembrane domain and kappa light chain sequences (**Supplementary Figure S1B**). This was also constructed by VectorBuilder.

#### 2.1.3 Control plasmids

Negative control plasmids were also constructed by VectorBuilder. The T cell negative control had an EF1α promoter and EGFP. The B cell negative control plasmid contained an EF1α promoter with dTomato.

### 2.2 Cell culture and isolation

Jurkat and Nalm6 cells (obtained from ATCC, Manassas, VA) were cultured in RPMI 1640 (Hyclone, Logan, UT) with 10% fetal bovine serum (FBS) (Hyclone), 100 units/ml Penicillin (Hyclone), 100 ug/ml Streptomycin (Hyclone), and 0.25 ug/ml Amphotericin B (Sigma-Aldrich, St. Loius, MO) at 37°C and 5% CO_2_.

Primary human T cells were obtained from healthy human donors (IRB#: IRB2023-130). Whole blood was collected into Vacutainer^®^ CPT™ tubes (BD Biosciences, Franklin Lakes, NJ) with sodium heparin and spun to isolate PBMCs, following the manufacturer’s instructions. T cells were then isolated using the EasySep human T cell isolation kit (StemCell, Vancouver, BC), following the manufacturer’s instructions. Isolated Primary T cells were cultured in RPMI 1640 (Hyclone) with 10% FBS (Hyclone), 1% Glutamax (Gibco, Waltham, MA), 10mM HEPES (Hyclone), 100 units/ml Penicillin, 100 ug/ml Streptomycin, and 0.25 ug/ml Amphotericin B (Sigma-Aldrich) at 37°C and 5% CO_2_. Primary human T cell media was also supplemented with 100 IU/ml rhIL-2 (Miltenyi, Bergisch Gladbach, Germany). T cells were activated/expanded with anti-CD3/anti-CD28 paramagnetic beads (Thermo Fisher Scientific, Waltham, MA) at a 3:1 bead-to-cell ratio.

293FT cells were cultured in D-MEM (Hyclone) with 10% FBS (Hyclone), 0.1 mM MEM non-essential amino acids (NEAA) (Gibco), 6mM L-glutamine (Hyclone), 1mM sodium pyruvate (Gibco), 100 units/ml Penicillin (Hyclone), 100 ug/ml Streptomycin (Hyclone), 0.25 ug/ml Amphotericin B (Sigma), and 500 mg/ml Geneticin (Thermo Fisher Scientific) at 37°C and 5% CO2. At 80% confluency, the cells were passaged to maintain optimal growth rates.

### 2.3 Lentivirus production

All previously described plasmid backbones were third-generation lentiviral backbones. To produce lentivirus, low passage 293FT cells were co-transfected with the transfer plasmid, lentiviral packaging, and envelope plasmids (pMD2.g, pRSV, and PMDL) using Lipofectamine 3000™ as previously described (22). Lentiviral supernatant was collected, filtered, and ultracentrifuged following the previously mentioned protocol. 293FT cells were then transduced with varying amounts of virus to titer the lentivirus. Multiplicity of infection (MOI) in primary human T cells was calculated following a similar transduction process based on the previously determined titer.

### 2.4 Lentiviral transduction

#### 2.4.1 Cell line transduction and sorting

To create CAAR/control cell lines in Jurkat cells, and for M22/K1-18/control cell lines in Nalm6 cells, Jurkat or Nalm6 cells were transduced with lentivirus at an MOI of 2 with 8 ug/ml Polybrene (Millipore Sigma, Burlington, MA). After 24 hours of co-culture, the cells were centrifuged (300xg) and fresh media was replaced. 48 hours after transduction, cells were fluorescence-activated cell sorted (FACS) for expression of their respective fluorescent reporter genes and then cryopreserved.

#### 2.4.2 Primary human T cell transduction and expansion

Primary human T cells were isolated and cultured as previously described. After 24h of activation with CD3/CD28 beads, the T cells were transduced with lentivirus at a MOI of 2 with 8ug/ml Polybrene (Millipore Sigma). 24h later, the cells and beads were centrifuged (300xg) and replaced with fresh media supplemented with rhIL-2. The cells were split about every 2 days to maintain a cell density of 1-2 x 10^6^ cells/ml. Cells were expanded for 7 days before removal of the beads, following the manufacturer’s instructions. At this point, a small portion of the cells were evaluated for their transduction efficiency (**Supplementary Figure S1D**) using flow cytometry (CytoFLEX, Beckman Coulter, Brea, CA) to measure the expression of the fluorescent reporter. It was previously validated that fluorescent reporter expression was consistent with the surface expression of the CAAR. After bead removal, the cells were rested in media without IL-2 supplementation for 24 hours before beginning functional assays.

### 2.5 Anti-TSHR Ab and TSH binding analysis in Jurkat cells

For the initial binding test with anti-TSHR Ab, sorted TSHR CAAR T cells and GFP Jurkat cells were co-incubated with saturating conditions of anti-TSHR Ab (M22) (Cell Sciences, Newburyport, MA) on ice. A secondary stain, goat anti-human IgG1: PE (Invitrogen, Waltham, MA), was then added in saturating conditions on ice. Samples were run using flow cytometry (CytoFLEX, Beckman Coulter) and analyzed in FlowJo 10 (FlowJo, Ashland, OR). The anti-TSHR binding titration was performed as described above but with varying amounts of M22 Ab. Gating scheme shown (**Supplementary Figure S1E**). Data was analyzed using FlowJo 10 and Prism 7.0 (GraphPad, Boston, MA).

TSH binding titrations were performed using the same protocol as described above with a His-tagged rhTSH (Acro Biosystems, Newark, DE) and a mouse anti-His: PE Ab (BioLegend, San Diego, CA). Gating scheme shown (**Supplementary Figure S1G**).

### 2.6 Activation experiments in Jurkat cells

24-well tissue culture-treated plates were coated with 10ug/ml anti-TSHR (M22) Ab at 4°C overnight. They were then washed twice with PBS before adding Jurkat CAAR T cells. 0.5 x 10^6^ cells were added to the wells and incubated for 24h. Cells were then stained with mouse anti-human CD69: PerCP-Cy5.5 Ab (Biolegend) on ice. After washing, they were run on the flow cytometer and analyzed using FlowJo 10 and Prism 7.0.

### 2.7 Cytotoxicity experiments

#### 2.7.1 Flow cytometry cytotoxicity analysis

To evaluate the cytotoxicity of the CAAR T cells, we used a co-culture method with primary human T cells (either control or CAAR T cells) and the target B cell lines (M22 or K1-18 GD B cell lines or control Nalm6 dTomato (n6dt) B cells), each in combination with one of the other cell types. Each well contained 10,000 B cells and 10,000 CAAR T cells in 200ul of primary human T cell media, as described above, without IL-2 supplementation. Plates were made for each time point and run on the flow cytometer using volumetric acquisition to count the CAAR T cells and the B cells. The gating scheme is shown (**Supplementary Figure S1H**). Data was analyzed using FlowJo 10 and Prism 7.0.

#### 2.7.2 Cytometric Bead Array

Cytokine levels were measured using Cytometric Bead Array (CBA) flex sets (BD Biosciences) for human IFNγ, IL-2, IL-6, and TNF. Supernatants from cytotoxicity assays described above were saved at 24h and frozen at -20°C for up to 3 months. The supernatant was thawed at room temperature, then the protocol was followed according to manufacturer instructions. Data was analyzed using FlowJo 10 and Prism 7.0.

#### 2.7.3 Proliferation assay

CellTrace Violet (Thermo Fisher Scientific) was used for a proliferation analysis of the CAAR T cells. Primary CAAR T cells were stained with CellTrace Violet following manufacturer instructions. They were then co-cultured with B cells as described previously. Analysis was performed using FlowJo’s proliferation software. CellTrace data from time 0 was used to set the baseline for the samples, and the proliferation index from FlowJo’s software at time 72 was used in statistical analysis, performed in Prism 7.0.

### 2.8 Plasma source patient characteristics

Healthy plasma was collected from a healthy donor (IRB2023-130). GD Plasma samples were purchased through PlasmaLab International (Everett, WA). Plasma sample patient characteristics are described in **Table 1**.

**Table 1:** GD plasma sample patient characteristics.

	GD diagnosis	Gender	Age	TRAb titer	Ethnicity
Healthy plasma	–	M	22	0.0 IU/L	Caucasian
GD plasma 1	+	F	51	1.05 IU/L	Caucasian
GD plasma 2	+	F	57	3.54 IU/L	Caucasian
GD plasma 3	+	M	32	7.36 IU/L	Other

## 3 Results

### 3.1 CAAR T cells designed with varying epitopes of TSHR as the binding domain of the CAAR bind to anti-TSHR autoantibody and are activated

**Figure 1:**
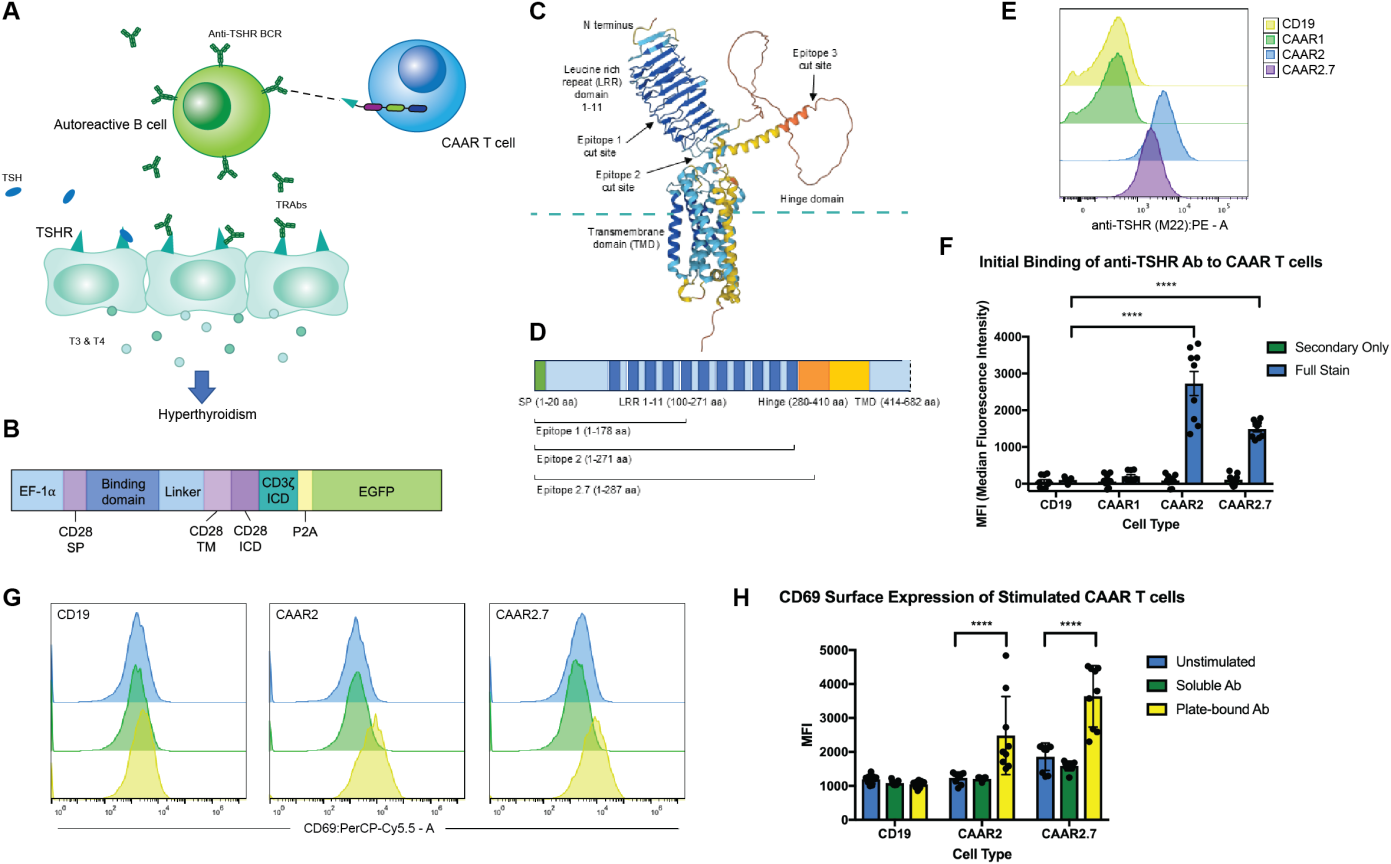
CAAR T cell design, TSHR epitope selection, and binding and activation. **(A)** Schematic depicting TSHR CAAR T cell specifically targeting autoreactive B cells in GD via their anti-TSHR BCRs. **(B)** CAR and CAAR construct schematic illustrating the promoter, binding domain location, transmembrane and activation domains, and EGFP for visualization (SP = signal peptide, TM = transmembrane, ICD = Intracellular domain). **(C)** Representative model of TSHR using AlphaFold 2 showing the key domains. **(D)** TSHR gene map showing the epitopes selected for the binding domains of the CAARs. **(E)** Representative flow cytometry graphs of anti-TSHR autoantibody (M22) binding to CAAR constructs. **(F)** CAAR2 and CAAR2.7 significantly bound to M22 Ab, while CAAR1 did not. Compared to a CD19 CAR for a control. (t-test, n=9, ****p<.0001). **(G)** Representative flow cytometry graphs showing activation of CAAR constructs after 24h stimulation with soluble or plate-bound M22 Ab. **(H)** Plate-bound M22 Ab significantly activated CAAR2 and CAAR2.7 after 24h of stimulation as measured by CD69 expression, though soluble M22 Ab did not significantly activate either CAAR (t-test, n=9, ****p<.0001).

To generate a CAAR construct for GD, we used TSHR as the binding domain, allowing for specific targeting of anti-TSHR B cells (**Figures 1A, B**). TSHR has an extracellular domain comprising a leucine-rich repeat (LRR) domain as well as a hinge domain which connects it to the transmembrane and intracellular domains of the protein (**Figure 1C**). The LRR is the key binding domain we needed to include, as this is where both TSH and stimulating anti-TSHR antibodies (TRAbs) bind (12, 23). GD patients can have blocking and neutral TRAbs as well, which typically bind to the hinge domain or N-terminus (24). When selecting TSHR epitopes to serve as the binding domain for our TSHR CAAR, we prioritized including as much of the LRR domain as possible, while selecting various sizes to test for proper surface expression of TSHR. We modeled multiple epitopes of TSHR using AlphaFold 2 to predict if the cutoff points affect their structure and folding (**Figure 1C**). Epitope 3 (which included the entirety of the hinge domain) was modified to Epitope 2.7 based on predictive modeling to optimize the folding of TSHR (**Figure 1D**). Epitopes 1, 2, and 2.7 were selected and cloned into our CAR construct.

When the CAAR constructs were transduced into Jurkat cells, we found that CAAR2 and CAAR2.7 showed significant binding to a patient-derived, stimulating monoclonal anti-TSHR Ab (M22) (**Figures 1E, F**). CAAR1 did not show any detectable binding to the M22 Ab, potentially because it does not include the entire LRR domain, preventing complete binding of the M22 Ab or causing improper folding of the LRR domain; thus, CAAR1 was excluded from further experiments. We then evaluated the activation of our CAAR T cells when stimulated with soluble and plate-bound M22 Ab. Neither CAAR2 nor CAAR2.7 were significantly activated by soluble M22 Ab; however, plate-bound M22 Ab did significantly activate both CAAR2 and CAAR2.7, as measured by CD69 expression (**Figures 1G, H**). Plate-bound Ab mimics a cell-to-cell interaction allowing for immunological synapse formation and full activation of a CAR T cell, which is consistent with what we observed.

### 3.2 TSHR CAAR T cells specifically eliminate autoreactive anti-TSHR B cells but leave other B cells untouched

**Figure 2:**
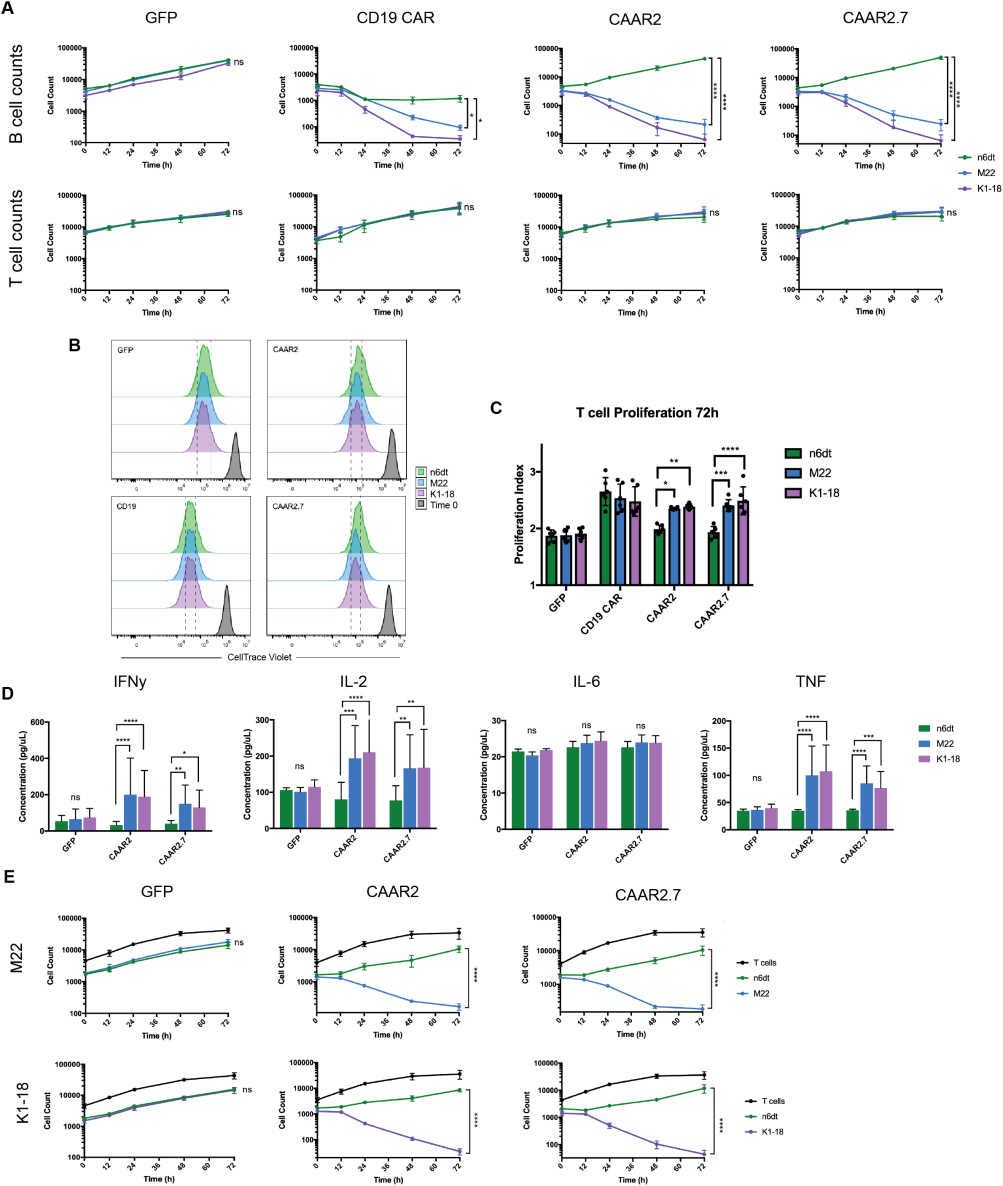
TSHR CAAR T cells exhibit cytotoxicity against anti-TSHR B cells, but not normal B cells. **(A)** B cell counts measured by flow cytometry showed that CAAR2 and CAAR2.7 eliminated anti-TSHR B cells by 72 hours, while normal B cells (Nalm6 dTomato (n6dt)) grew as expected. GFP T cells did not eliminate any B cells and CD19 CAR T cells eliminated most B cells (t-tests at 72h, n=6, *p<.05, **p<.01, ***p<.001, ****p<.0001). There was no significant difference in T cell counts (t-tests at 72h, n=6) **(B)** Representative flow cytometry graphs showing T cell proliferation measured by CellTrace Violet. Differences in the histograms are subtle, so dashed lines are included to make differences clearer. **(C)** Quantified proliferation index for CAAR2 and CAAR2.7 shows a significantly higher proliferation index when co-cultured with anti-TSHR B cells than with normal B cells, matching the difference in levels between GFP T cells and the CD19 CAR T cells (ANOVA multiple comparisons, n=6, *p<.05, **p<.01, ***p<.001, ****p<.0001). **(D)** CAAR2 and CAAR2.7 released significantly more IFNγ, IL-2, and TNF when co-cultured with anti-TSHR B cells compared with normal B cells, measured by cytometric bead array after 24h (ANOVA multiple comparisons, n=6, *p<.05, **p<.01, ***p<.001, ****p<.0001). **(E)** When co-cultured with a 1:1 ratio of normal to anti-TSHR B cells, CAAR2 and CAAR2.7 eliminated the anti-TSHR B cells by 72 hours while the normal B cells were unaffected (t-tests at 72h, n=6, ****p<.0001).

To evaluate the cytotoxic ability of the TSHR CAAR T cells, we used lentivirus to transduce the CAAR constructs into primary human T cells and then co-cultured them with normal and anti-TSHR B cells. Anti-TSHR B cell lines were developed by engineering synthetic BCRs with anti-TSHR variable region sequences derived from GD patients and shown to be pathogenic and from the stimulating class of anti-TSHR Abs. Two anti-TSHR B cell lines were developed, one with a stimulating anti-TSHR BCR (M22) and one with a blocking anti-TSHR BCR (K1-18)(**Supplementary Figures S1A-C**). Stimulating anti-TSHR BCRs are the primary pathogenic autoantibodies in GD, but we also wanted to determine if the TSHR CAAR T cells would be capable of eliminating blocking anti-TSHR B cells as well. We then co-cultured each T cell variant (CAAR2, CAAR2.7, a CD19 CAR as a positive control, and T cells transduced with only EGFP as a negative control) with each B cell variant independently (a Nalm6 dTomato (n6dt) “normal” control, and the two anti-TSHR B cell lines, M22 and K1-18). In the presence of the GFP T cells, the B cells all grew normally, and the CD19 CAR T cell positive control eliminated most of the B cells they were co-cultured with (**Figure 2A**). By 72 hours, CAAR2 and CAAR2.7 eliminated the anti-TSHR B cells, but the n6dt B cells grew normally (**Figure 2A**). The T cells all proliferated as expected, and there was no significant difference in the number of T cells by 72 hours (**Figure 2A**). However, when the T cell’s proliferation was evaluated using CellTrace, we found that the proliferation index of CAAR2 and CAAR2.7 was significantly higher when co-cultured with anti-TSHR B cells compared to normal n6dt B cells, which matched the proliferation index of the CD19 CAR compared to the GFP T cells (**Figures 2B, C**). We also measured the levels of proinflammatory cytokines secreted at 24 hours. CAAR2 and CAAR2.7 secreted significantly more IFNγ, IL-2, and TNF when co-cultured with anti-TSHR B cells compared to normal n6dt B cells, whereas IL-6 levels were not different (**Figure 2D**). We evaluated if there would be significant “bystander” killing of normal n6dt B cells when the CAAR T cells were co-cultured with a mix of normal and anti-TSHR B cells. The CAAR T cells still eliminated the anti-TSHR B cells by 24 hours, but the normal n6dt B cell numbers were similar to the negative control (GFP-only T cells) (**Figure 2E**).

### 3.3 Soluble anti-TSHR Ab binds to TSHR CAAR T cells; CAAR2 is not significantly affected by soluble anti-TSHR Ab while CAAR2.7 cytotoxicity and cytokine production is significantly affected

**Figure 3:**
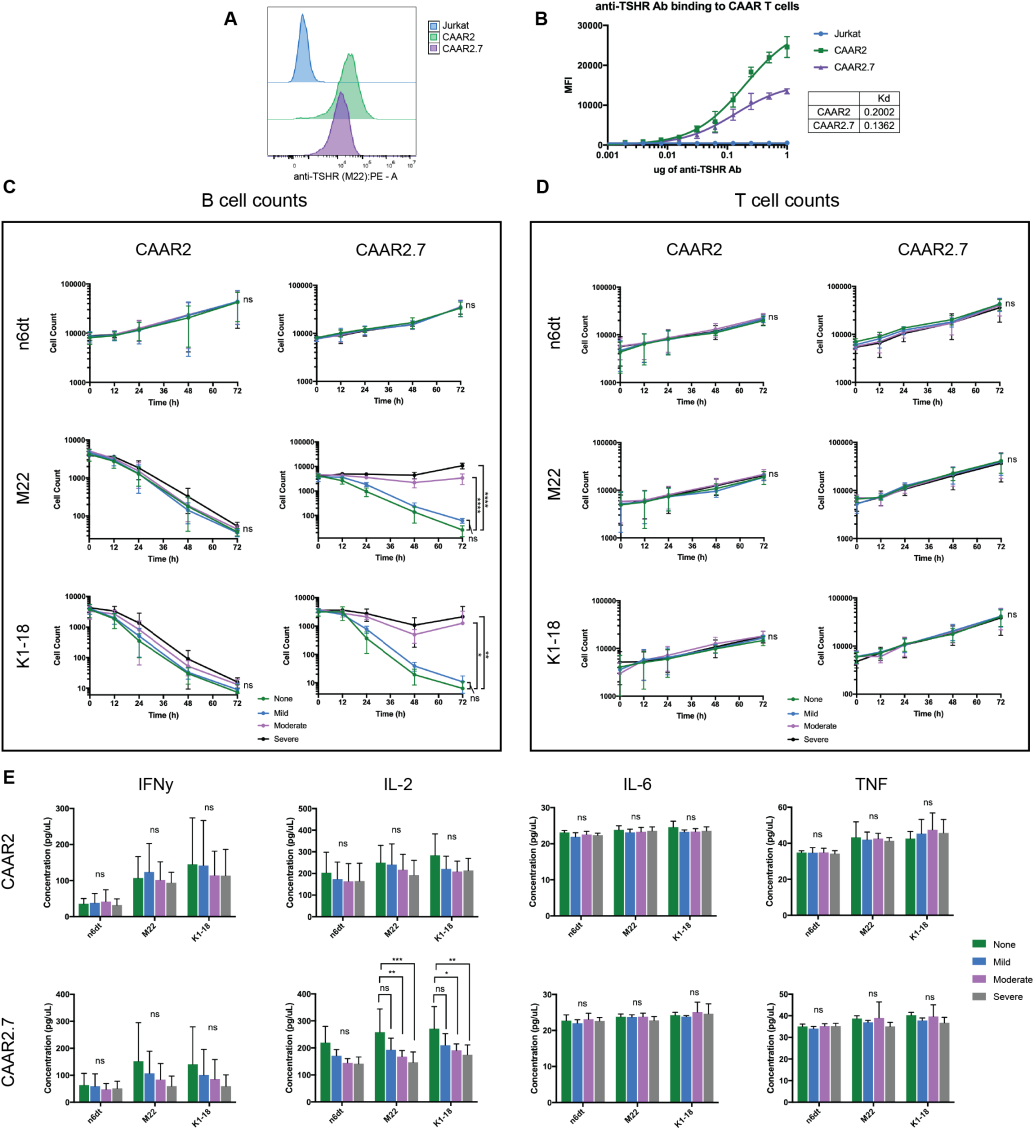
Soluble anti-TSHR Ab binds to CAAR2 and CAAR2.7, but only CAAR2.7 is significantly affected by its presence. **(A)** Representative flow cytometry binding showing anti-TSHR Ab (M22) binding to Jurkat cells, CAAR2, and CAAR2.7 at a concentration of 1ug M22 Ab. **(B)** Anti-TSHR Ab (M22) binding titration to Jurkat CAAR T cells and untransduced Jurkat cells. CAAR2 has a higher Kd than CAAR2.7, implying that CAAR2.7 has a higher binding affinity to anti-TSHR Ab, though CAAR2 has more receptors available to bind, as it has a higher B_max_: CAAR2 B_max_=30345 MFI, CAAR2.7 B_max_=15677 MFI. **(C)** B cell counts measured by flow cytometry show that CAAR2 was not significantly affected by the presence of anti-TSHR Ab, in biological levels characteristic of mild, moderate, and severe GD (1.75 IU/L, 10IU/L, 40 IU/L), and was able to eliminate anti-TSHR B cells by 72 hours. CAAR2.7 was significantly less effective at eliminating anti-TSHR B cells in the presence of moderate and severe levels of anti-TSHR Ab, though killing is still happening (t-tests at 72h, n=6, *p<.05, **p<.01, ****p<.0001). **(D)** There were no significant differences in T cell counts over time with the addition of soluble anti-TSHR Ab (t-tests at 72h, n=6). **(E)** CAAR2 did not have any significant difference in cytokine levels, measured by CBA after 24 hours. CAAR2.7 had significantly lower levels of IL-2 when co-cultured with anti-TSHR B cells with moderate and severe levels of anti-TSHR Ab compared to the levels of IL-2 when CAAR2.7 was with only anti-TSHR B cells (ANOVA multiple comparisons, n=6, *p<.05, **p<.01, ***p<.001).

Our initial binding experiments showed that anti-TSHR Ab (M22) bound to CAAR2 and CAAR2.7 (**Figure 1F**). We performed a binding titration and found that CAAR2.7 has a slightly higher binding affinity to anti-TSHR Ab (M22), as shown by its lower Kd value (CAAR2 Kd = .2002 mol/L, CAAR2.7 Kd = .1362 mol/L) (**Figures 3A, B**). We also determined that CAAR2 has more receptors expressed and available to bind than CAAR2.7 as it has a higher B_max_, representing the maximum binding capacity (CAAR2 B_max_=30345 MFI, CAAR2.7 B_max_=15677 MFI) (**Figures 3A, B**). One concern of the CAAR system is that the binding of soluble anti-TSHR Ab to the CAAR could overstimulate the CAAR T cells and cause excessive proinflammatory cytokine release, leading to cytokine release syndrome (CRS). Another concern is that the binding of soluble Ab to the TSHR binding domains could prevent activation of the CAAR T cells and removal of the autoreactive B cells. To evaluate these possibilities, we performed the cytotoxicity and cytokine release assays as previously mentioned in the presence of mild, moderate, and severe GD anti-TSHR Ab levels (1.75 IU/L, 10IU/L, 40 IU/L) along with a no Ab control. We observed that increasing levels of anti-TSHR Ab did not significantly affect the cytotoxicity of CAAR2 (**Figure 3C**). There was also no significant difference in cytokine levels produced by CAAR2 with the different levels of anti-TSHR Ab present (**Figure 3E**). However, the cytotoxicity of CAAR2.7 was inhibited in a dose-dependent manner with the addition of anti-TSHR Ab, though there was still killing of anti-TSHR B cells occurring (**Figure 3C**). CAAR2.7 also released significantly less IL-2 in the presence of moderate and severe levels of anti-TSHR Ab (**Figure 3E**). For both CAAR2 and CAAR2.7, there was no significant difference in T cell counts over time with the addition of soluble anti-TSHR Ab (**Figure 3D**).

### 3.4 Soluble TSH does not bind to the TSHR CAAR T cells and does not significantly affect their cytotoxicity and cytokine production

**Figure 4:**
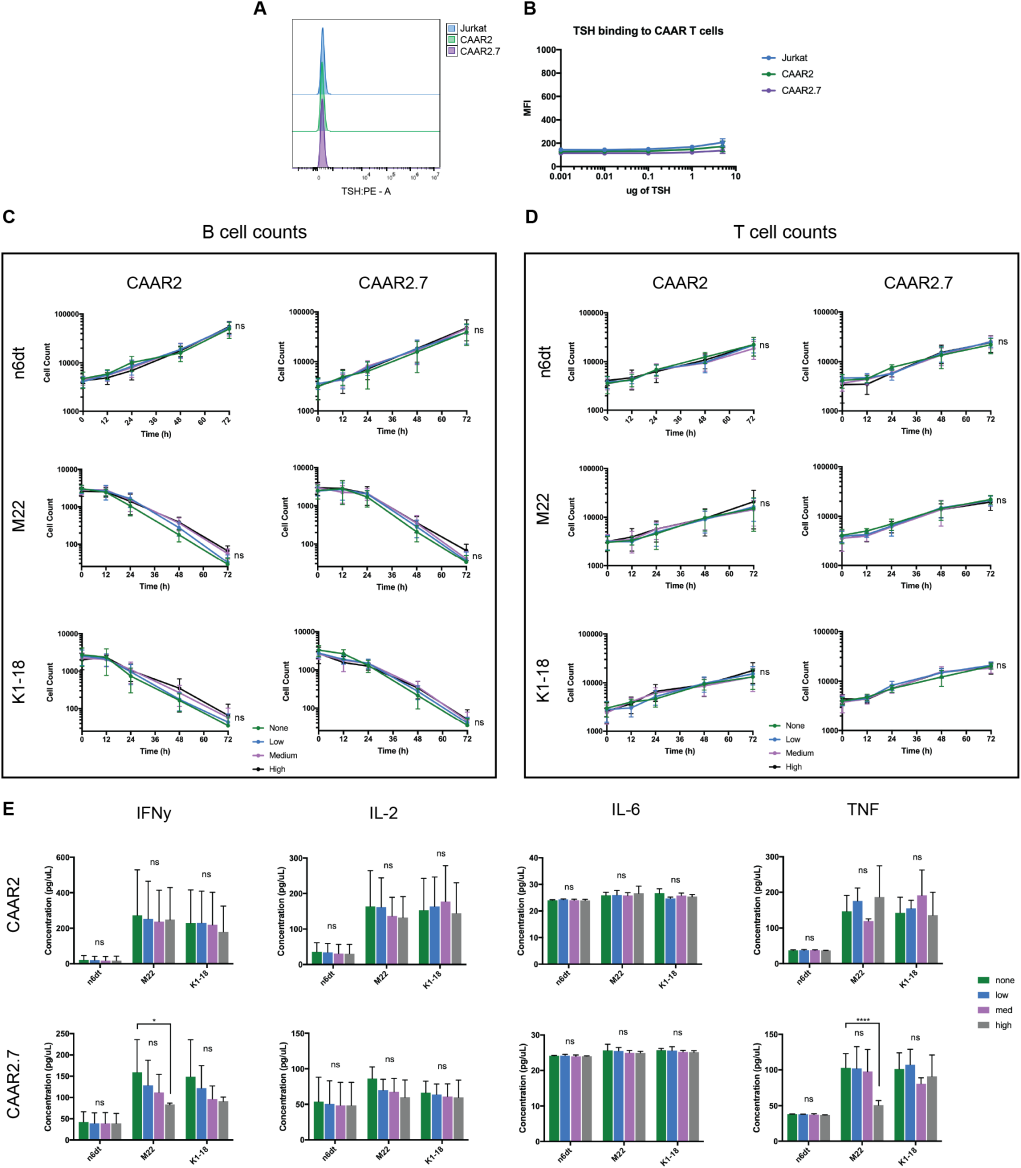
TSH does not significantly bind to TSHR CAAR T cells and does not affect their cytotoxicity. **(A)** Representative flow cytometry binding showing TSH does not bind to CAAR T cells. **(B)** TSH binding titration to Jurkat CAAR T cells and untransduced Jurkat cells shows that TSH does not significantly bind to the CAAR T cells, even at 5ug/100ul, which is over 250x the top of the biological range (t-tests, n=9). **(C)** Both CAAR2 and CAAR2.7 in the presence of biological levels of TSH, the low, mid, and high points of the normal range (.5mIU/L, 2mIU/L, 5mIU/L), do not show differences in B cell counts, signifying their cytotoxicity is unaffected by the presence of TSH (t-tests at 72h, n=6). **(D)** T cell counts over time were also unaffected by the presence of TSH (t-tests at 72h, n=6). **(E)** Cytokine levels for CAAR2 were not significantly affected by the presence of TSH. CAAR2.7 only showed significant differences in IFNγ and TNF production when co-cultured with M22 B cells and high levels of TSH (ANOVA multiple comparisons, n=6, *p<.05, ****p<.0001).

As the binding domain of our TSHR CAAR T cells are epitopes of TSHR, and thus the receptor for TSH, it was likely that TSH could bind to our CAAR T cells and have an impact on their cytotoxicity, as soluble anti-TSHR Ab did. We performed a binding titration of TSH with the CAAR T cells and found that TSH did not bind significantly to the CAAR T cells, even at much higher levels of TSH than is biologically relevant (**Figures 4A, B**). We also evaluated the cytotoxicity of our CAAR T cells against anti-TSHR B cells in the presence of biologically relevant levels of TSH: the low and high points of the normal range, as well as a midpoint (.5mIU/L, 2mIU/L, 5mIU/L). We observed that TSH did not significantly affect the cytotoxicity of CAAR2 or CAAR2.7, nor did it impact T cell counts (**Figures 4C, D**). When evaluating the effect of soluble TSH on the cytokine release of the CAAR T cells, we found that CAAR2 did not have any significant difference with the addition of TSH. CAAR2.7 was only significantly affected by high levels of TSH, where we see that IFNγ and TNF levels were significantly lower with the M22 anti-TSHR B cell line only (**Figure 4E**).

### 3.5 In GD patient plasma, both CAAR2 and CAAR2.7 exhibit cytotoxicity against anti-TSHR B cells, though CAAR2.7 is less efficient in patient plasma compared to healthy plasma

**Figure 5:**
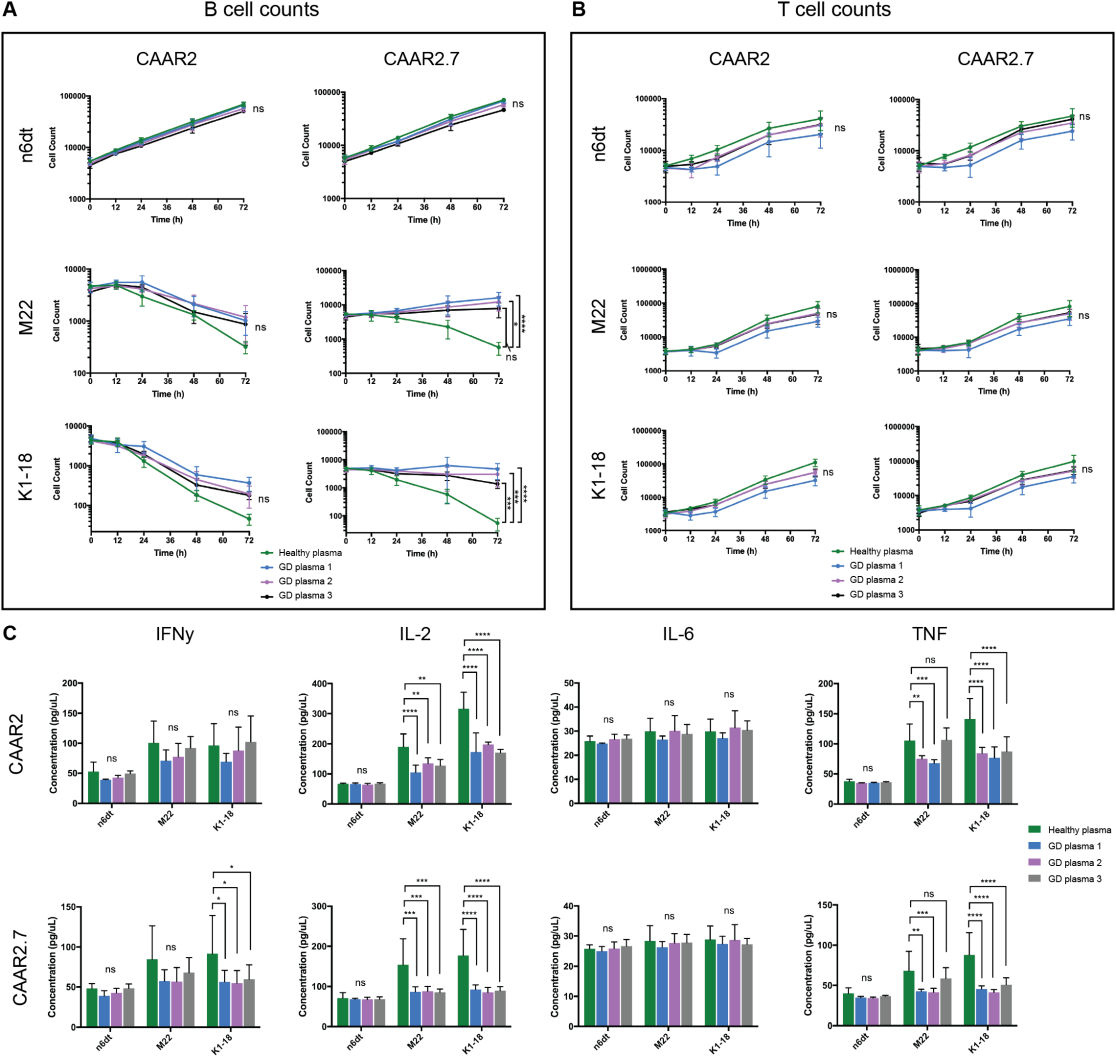
CAAR2 and CAAR2.7 cytotoxicity and cytokine release when co-cultured in GD plasma. **(A)** CAAR2 did not eliminate significantly more anti-TSHR B cells in the presence of GD plasma compared to healthy plasma, and the plasma did not affect its specificity towards the anti-TSHR B cells. CAAR2.7 was significantly less efficient at killing anti-TSHR B cells in the presence of GD plasma than healthy plasma but did not exhibit any cytotoxicity against healthy B cells in the presence of the plasma (t-tests at 72h, n=6, *p<.05, ***p<.001, ****p<.0001). **(B)** There were no significant differences in T cell counts when co-cultured with B cells in the different plasmas (t-tests at 72h, n=6). **(C)** Compared with healthy plasma, CAAR2 secretes significantly less IL-2 and TNF when co-cultured with anti-TSHR B cells with GD plasma. CAAR2.7 secretes significantly less IFNγ when co-cultured with K1-18 B cells with GD plasma compared with healthy plasma and also secretes significantly less IL-2 and TNF when co-cultured with either anti-TSHR B cell line in the presence of GD plasma compared with healthy plasma (ANOVA multiple comparisons, n=6, *p<.05, **p<.01, ***p<.001, ****p<.0001).

To evaluate our TSHR CAAR T cells in a biological context, we obtained plasma from GD patients with varying titers of anti-TSHR Abs and divergent disease severity (**Table 1**). We evaluated the cytotoxicity of our CAAR T cells in the plasma of three GD patients as well as in healthy donor plasma. CAAR2 did not have any significant difference in cytotoxicity against anti-TSHR B cells between the healthy plasma and any of the diseased plasma samples, though trends suggest the healthy serum seems to be slightly more conducive to CAAR T cell elimination of anti-TSHR B cells (**Figure 5A**). However, CAAR2.7 did have significantly less killing of anti-TSHR B cells with the GD plasma samples compared to the healthy plasma. The T cell counts do not have any significant differences between the different plasma samples, though it is interesting to note that the T cell growth rate shows trends of slower growth depending on which plasma the cells were grown in (**Figure 5B**). When evaluating the cytokine production of the TSHR CAAR T cells, we observed that CAAR2 was less affected by the GD plasma than CAAR2.7 (**Figure 5C**). CAAR2 showed significantly less IL-2 and TNF production in the GD plasma compared with healthy plasma, and CAAR2.7 showed significantly less production of IFNγ, IL-2, and TNF in the GD plasma.

## 4 Discussion

Here we report the generation of several novel TSHR CAAR T cells that can specifically bind to anti-TSHR Abs and GD autoreactive B cells via their anti-TSHR BCR. Both CAAR2 and CAAR2.7 exhibit significant cytotoxicity against anti-TSHR B cells, and their proliferation and cytokine production are typical of functional CAR T cell therapies (**Figure 2**). The ability to eliminate autoreactive B cells in GD and thus reduce or deplete autoantibodies is key for stopping the most common condition of GD, hyperthyroidism. However, unlike any other treatments currently available, specific elimination of autoreactive B cells can also stop Graves’ Eye Disease and cardiomyopathy that are caused by cross-reactivity of anti-TSHR autoantibodies with the eyes and heart, while avoiding compromising the immune system.

While we were working on this research, a paper was released with a similar goal of using TSHR as a binding domain for CAAR T cells as a treatment for GD (25). This paper shows that their TSHR CAAR T cell effectively eliminated a mouse anti-TSHR hybridoma *in vitro* and *in vivo* (25). This is an important finding and provides additional evidence to support the possibility of TSHR CAAR T cells as a treatment for GD. However, our work examines and addresses several critical aspects that were not addressed in the previous study. First, in their paper, they only use one CAAR construct with the entire extracellular portion of TSHR as their binding domain, which includes the LRR and hinge domains. Following our modeling of different epitopes of TSHR, we selected several epitopes to serve as our binding domains, and the ultimately successful ones were CAAR2 (just the LRR) and CAAR2.7 (the LRR and part of the hinge region), which are both different from the epitope used in their paper. Additionally, we chose to use a human-based *in vitro* model and GD patient plasma to see how the CAAR T cells would perform in a more biologically relevant model for clinical application. The previous paper also did not address how soluble anti-TSHR Ab may affect the function of the CAR T cells, which is crucial considering the similarity of anti-TSHR Abs and anti-TSHR BCRs, the target of this therapy. Additionally the previous paper did not test the CAR T cells with patient serum as we did to evaluate their efficacy in a clinically relevant setting.

The slight differences in amino acid length of TSHR between CAAR2 and CAAR2.7 proved to have significant effects on CAAR T cell function when in the presence of soluble anti-TSHR Ab, TSH, and GD patient plasma. The cytotoxicity of CAAR2 was not significantly affected by the presence of soluble anti-TSHR Ab or GD plasma while CAAR2.7 was, making CAAR2 a prime choice for further development as a therapeutic for GD (**Figure 3**). CAAR2 does not include the hinge region of TSHR, while CAAR2.7 includes a portion of the hinge region. It is likely that the differences in the length of the TSHR hinge region included in the binding domain caused slight but critical structural differences in the LRR of TSHR, causing the different cytotoxicity effects induced by the presence of soluble anti-TSHR Ab and GD plasma. The slight structural differences between the two TSHR CAAR T cells and native TSHR could also be influencing why TSH does not seem to bind to the TSHR CAAR T cells in our binding titration (**Figure 4**). Our data on the different epitopes of TSHR as the binding domain of the CAAR T cells suggests that, in different disease settings, changing the epitope of the autoantigen binding domain even slightly can have significant effects on its efficacy. Future CAAR T cell therapies across different diseases would likely benefit from screening additional versions of the binding domain to optimize function.

We note the inherent limitations of the *in vitro* model used during this study but maintain that it still shows the promise of TSHR CAAR T cells as a possible treatment for GD. Nalm6 cells are not entirely representative of all human B cells; however, because of the low percentage of autoreactive B cells among total B cells in GD patients, it was not feasible to isolate GD B cells to serve as the target cells for the experiments in this paper. We instead chose to use patient-derived and validated sequences for autoreactive anti-TSHR BCRs, and transduced them into Nalm6 cells as an *in vitro* model of GD. By using GD patient plasma, we hoped to get a better picture of how effective the TSHR CAAR T cells perform in a patient setting.

This human-based *in vitro* model of GD includes the primary pathogenic stimulating anti-TSHR Ab/BCR (M22), as well as a blocking anti-TSHR BCR (K1-18). CAAR2 and CAAR2.7 were equally effective at eliminating M22 GD B cells and K1-18 GD B cells. While a majority of GD patients suffer from hyperthyroidism as caused by stimulating anti-TSHR Abs, a smaller subset of patients can exhibit hypothyroidism when their primary autoantibodies are blocking anti-TSHR Abs that prevent normal binding of TSH (26). Our data suggests that TSHR CAAR T cells could be an effective therapy for both GD-caused hyperthyroidism and GD-caused hypothyroidism.

CAR T cells have been remarkably effective in hematological malignancies, with many of their drawbacks being in the solid tumor area, which should not apply to this CAAR T cell application to GD. Cytokine release syndrome (CRS) is a negative potential side effect of CAR T cell therapies; however, in theory, there is a reduced risk of CRS because GD has significantly fewer autoreactive B cells compared to malignant cells in B cell cancers. Lymphodepleting chemotherapy prior to CAR T cell treatment would likely be necessary in CAAR T cell treatment for GD and is an additional risk to the patient for infections and other side-effects. While lymphodepletion prior to CAR T cell therapy is currently necessary for CAR T cells to be effective, research is being done to mitigate the limitations of this step in the CAR T cell process (27). The cost of CAR T cell therapies can be prohibitive as well. Though GD is not often life-threatening, lifelong treatment of GD, which typically requires hormone replacement therapy along with additional care, can be a great cost burden, which may justify the price of CAAR T cell therapy. Patients who suffer from Graves’ Eye Disease or cardiomyopathy, which do not have effective treatment options, would also benefit from TSHR CAAR T cell therapy, and the cost may be more justified in these cases. Ongoing research into ways to reduce the cost of CAR T cell therapies will also be influential in making CAAR T cell therapies for autoimmune diseases more feasible (28, 29).

In conclusion, TSHR CAAR T cells show promise as a treatment for GD by specifically eliminating the autoreactive B cells that cause the disease. GD is also a valuable and tractable model for the use of CAAR T cells in treating autoimmune diseases. The key autoantigens are well characterized in GD which allows for an easier application of CAAR T cells. Many other B cell-mediated autoimmune diseases could be targets for future CAAR T cell therapies once this therapy mechanism is further validated *in vivo* and in clinical trials. A Phase 1 clinical trial is currently ongoing to evaluate CAAR T cell therapy as a treatment for Muscle-specific tyrosine kinase (MuSK) Myasthenia Gravis (NCT05451212), though the other currently ongoing clinical trials for CAR T cells in autoimmune disease are focused on anti-CD19 and anti-BCMA CAR T cells. We believe that CAAR T cell therapies are a promising option for treating B cell-mediated autoimmune diseases, and that they have potential benefits of safety and specificity compared with CAR T cells that cause total B cell depletion.

## Data Availability

The original contributions presented in the study are included in the article/**Supplementary Material**. Further inquiries can be directed to the corresponding author.
