# Supplementary material for: Chimeric autoantibody receptor T cells specifically eliminate Graves’ Disease autoreactive B cells
Source: Front Immunol. 2025 Apr 8;16:1562662. doi: 10.3389/fimmu.2025.1562662 (PMC12011768; doi:10.3389/fimmu.2025.1562662)
Supplement: Supplementary file 1 [file Table1.docx]

Supplementary Material

# Supplementary Figure

**
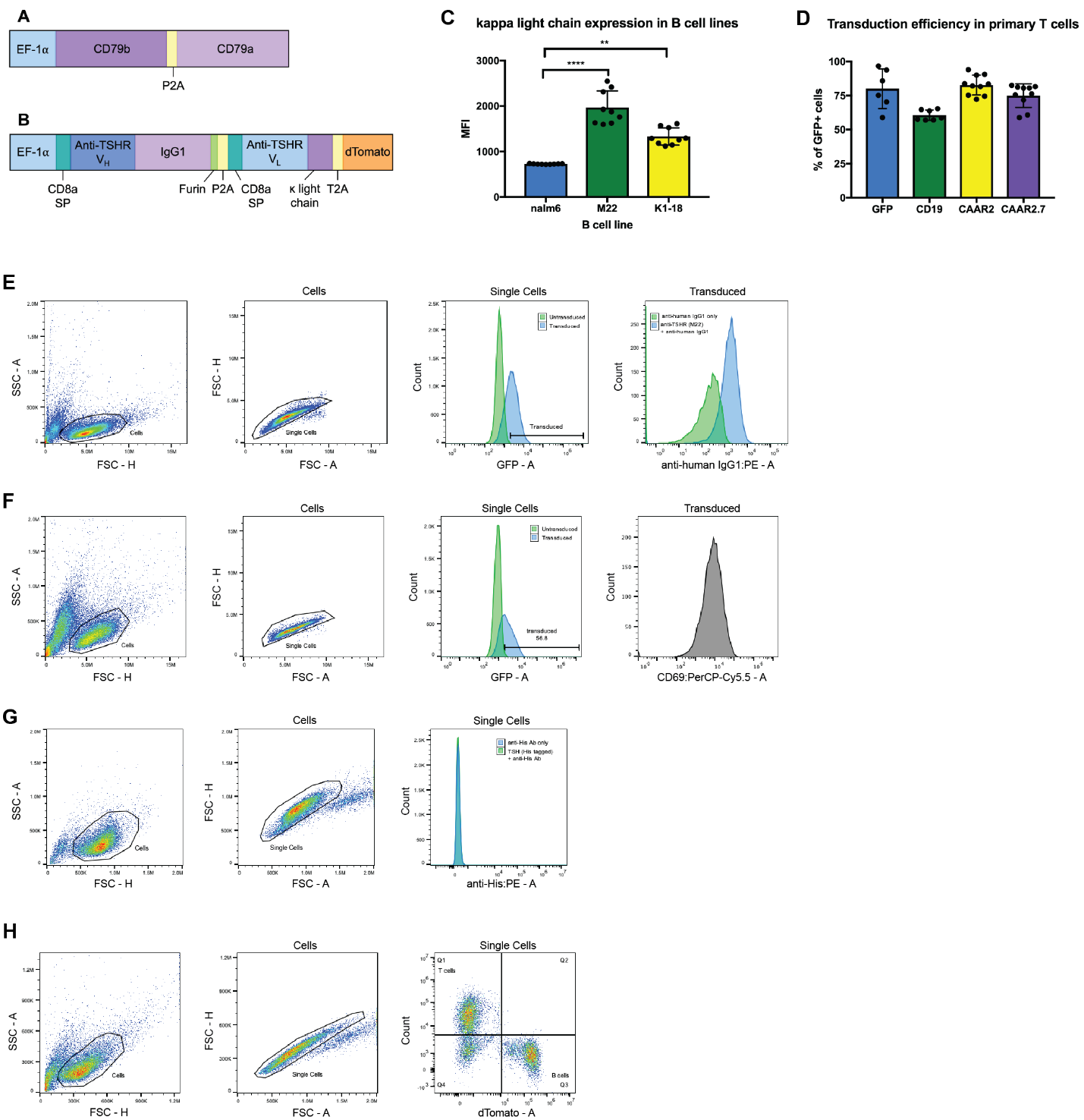
**

**Supplementary Figure 1.** A) B cell coreceptor insert schematic, with CD79a and CD79b. B) BCR schematic depicting the insert for the synthetic anti-TSHR BCRs that were transduced along with B cell co-receptor insert into nalm6 cells to form the anti-TSHR B cell lines used in the experiments. (VH = Variable heavy, VL = Variable light). C) Validation of the expression of the BCR on the anti-TSHR B cell lines using flow cytometry to measure the expression of the kappa light chain on the B cells. Nalm6 cells do not natively express any BCR or kappa light chain. These cells were later sorted for expression of the dTomato reporter and kappa light chain expression before use in further experiments (t-test, n=9, ***p<.001, ****p<.0001). D) Graph showing transduction rates of our GFP, CD19 CAR, and TSHR constructs in primary human T cells. The transduction rates were consistently above 70%. E) Flow cytometry gating scheme for anti-TSHR Ab (M22) binding to CAAR T cells. F) Flow cytometry gating scheme for activation, by CD69 expression, of CAAR T cells when stimulated with anti-TSHR Ab. G) Flow cytometry gating scheme for measuring TSH binding to CAAR T cells. H) Flow cytometry gating scheme for cytotoxicity experiments of CAAR T cells against B cells. T cells expressed EGFP, and B cells expressed dTomato.

# Supplementary Table

| Figure |  | Exact statistical values |
| --- | --- | --- |
| Figure 1 | F | CD19 vs. CAAR1 = .9262  CD19 vs. CAAR2 <.0001  CD19 vs. CAAR2.7 <.0001 |
|  | H | CD19- unstimulated vs. soluble Ab = .8940  CD19- unstimulated vs. plate-bound Ab = .8194  CAAR2- unstimulated vs. soluble Ab = .5154  CAAR2- unstimulated vs. plate-bound Ab <.0001  CAAR2.7- unstimulated vs. soluble Ab <.0001 |
| Figure 2 | A | B cell counts:  GFP- n6dt vs. M22 = .9486  GFP- n6dt vs. K1-18 = .1729  CD19- n6dt vs. M22 = .0226  CD19- n6dt vs. K1-18 = .0267  CAAR2- n6dt vs. M22 <.0001  CAAR2- n6dt vs. K1-18 <.0001  CAAR2.7- n6dt vs. M22 <.0001  CAAR2.7- n6dt vs. K1-18 <.0001  T cell counts:  GFP- n6dt vs. M22 = .7937  GFP- n6dt vs. K1-18 = .6583  CD19- n6dt vs. M22 = .9740  CD19- n6dt vs. K1-18 = .9129  CAAR2- n6dt vs. M22 = .6748  CAAR2- n6dt vs. K1-18 = .4913  CAAR2.7- n6dt vs. M22 = .7196  CAAR2.7- n6dt vs. K1-18 = .3695 |
|  | C | GFP- n6dt vs. M22 >.9999  GFP- n6dt vs. K1-18 >.9999  CD19- n6dt vs. M22 = .9819  CD19- n6dt vs. K1-18 = .7672  CAAR2- n6dt vs. M22 = .0114  CAAR2- n6dt vs. K1-18 = .0037  CAAR2.7- n6dt vs. M22 = .0002  CAAR2.7- n6dt vs. K1-18 <.0001 |
|  | D | IFNy:  GFP- n6dt vs. M22 = .9822  GFP- n6dt vs. K1-18 = .9357  CAAR2- n6dt vs. M22 <.0001  CAAR2- n6dt vs. K1-18 <.0001  CAAR2.7- n6dt vs. M22 = .0095  CAAR2.7- n6dt vs. K1-18 = .0403  IL-2:  GFP- n6dt vs. M22 = .9895  GFP- n6dt vs. K1-18 = .9716  CAAR2- n6dt vs. M22 = .0001  CAAR2- n6dt vs. K1-18 <.0001  CAAR2.7- n6dt vs. M22 = .0037  CAAR2.7- n6dt vs. K1-18 = .0043  IL-6:  GFP- n6dt vs. M22 = .4853  GFP- n6dt vs. K1-18 = .9096  CAAR2- n6dt vs. M22 = .1208  CAAR2- n6dt vs. K1-18 = .0872  CAAR2.7- n6dt vs. M22 = .0566  CAAR2.7- n6dt vs. K1-18 = .0811  TNF:  GFP- n6dt vs. M22 = .9951  GFP- n6dt vs. K1-18 = .9440  CAAR2- n6dt vs. M22 <.0001  CAAR2- n6dt vs. K1-18 <.0001  CAAR2.7- n6dt vs. M22 <.0001  CAAR2.7- n6dt vs. K1-18 = .0006 |
|  | E | M22:  GFP- n6dt vs. M22 = .2274  CAAR2- n6dt vs. M22 <.0001  CAAR2.7- n6dt vs. M22 <.0001  K1-18:  GFP- n6dt vs. K1-18 = .6400  CAAR2- n6dt vs. K1-18 <.0001  CAAR2.7- n6dt vs. K1-18 <.0001 |
| Figure 3 | B | CAAR2 goodness of fit- R squared = .9726  CAAR2.7 goodness of fit- R squared = .9618 |
|  | C | CAAR2:  n6dt- none vs. mild = .9975  n6dt- none vs. moderate = .9995  n6dt- none vs. severe >.9999  M22- none vs. mild >.9999  M22- none vs. moderate >.9999  M22- none vs. severe >.9999  K1-18- none vs. mild >.9999  K1-18- none vs. moderate >.9999  K1-18- none vs. severe >.9999  CAAR2.7:  n6dt- none vs. mild = .9657  n6dt- none vs. moderate = .9292  n6dt- none vs. severe = .9637  M22- none vs. mild = .9999  M22- none vs. moderate <.0001  M22- none vs. severe <.0001  K1-18- none vs. mild >.9999  K1-18- none vs. moderate >.9999  K1-18- none vs. severe >.9999 |
|  | D | CAAR2:  n6dt- none vs. mild = .8351  n6dt- none vs. moderate = .5059  n6dt- none vs. severe = .8870  M22- none vs. mild >.9999  M22- none vs. moderate = .6651  M22- none vs. severe = .9162  K1-18- none vs. mild = .7900  K1-18- none vs. moderate = .3283  K1-18- none vs. severe = .3953  CAAR2.7:  n6dt- none vs. mild = .9971  n6dt- none vs. moderate = .9449  n6dt- none vs. severe = .4293  M22- none vs. mild = .9926  M22- none vs. moderate = .9860  M22- none vs. severe = .8652  K1-18- none vs. mild = .9965  K1-18- none vs. moderate = .9999  K1-18- none vs. severe = .9469 |
|  | E | CAAR2:  IFNy:  n6dt- none vs. mild = .9998  n6dt- none vs. moderate = .9977  n6dt- none vs. severe = .9995  M22- none vs. mild = .9542  M22- none vs. moderate = .9977  M22- none vs. severe = .9755  K1-18- none vs. mild = .9996  K1-18- none vs. moderate = .7837  K1-18- none vs. severe = .7715  IL-2:  n6dt- none vs. mild = .8505  n6dt- none vs. moderate = .7020  n6dt- none vs. severe = .7189  M22- none vs. mild = .9949  M22- none vs. moderate = .8018  M22- none vs. severe = .4437  K1-18- none vs. mild = .3740  K1-18- none vs. moderate = .2364  K1-18- none vs. severe = .2890  IL-6:  n6dt- none vs. mild = .0854  n6dt- none vs. moderate = .6310  n6dt- none vs. severe = .3952  M22- none vs. mild = .4840  M22- none vs. moderate = .7060  M22- none vs. severe = .9363  K1-18- none vs. mild = .0636  K1-18- none vs. moderate = .0928  K1-18- none vs. severe = .2002  TNF:  n6dt- none vs. mild >.9999  n6dt- none vs. moderate >.9999  n6dt- none vs. severe = .9956  M22- none vs. mild = .9560  M22- none vs. moderate = .9934  M22- none vs. severe = .8609  K1-18- none vs. mild = .6958  K1-18- none vs. moderate = .2558  K1-18- none vs. severe = .5941  CAAR2.7:  IFNy:  n6dt- none vs. mild = .9996  n6dt- none vs. moderate = .9725  n6dt- none vs. severe = .9875  M22- none vs. mild = .6308  M22- none vs. moderate = .3079  M22- none vs. severe = .1120  K1-18- none vs. mild = .7061  K1-18- none vs. moderate = .4732  K1-18- none vs. severe = .1835  IL-2:  n6dt- none vs. mild = .1963  n6dt- none vs. moderate = .0219  n6dt- none vs. severe = .0156  M22- none vs. mild = .0508  M22- none vs. moderate = .0042  M22- none vs. severe = .0004  K1-18- none vs. mild = .0768  K1-18- none vs. moderate = .0130  K1-18- none vs. severe = .0023  IL-6:  n6dt- none vs. mild = .7107  n6dt- none vs. moderate = .9467  n6dt- none vs. severe = .9992  M22- none vs. mild = .9996  M22- none vs. moderate >.9999  M22- none vs. severe = .4947  K1-18- none vs. mild = .9351  K1-18- none vs. moderate = .6495  K1-18- none vs. severe = .9105  TNF:  n6dt- none vs. mild = .8910  n6dt- none vs. moderate = .9989  n6dt- none vs. severe = .9987  M22- none vs. mild = .6208  M22- none vs. moderate = .9969  M22- none vs. severe = .0917  K1-18- none vs. mild = .3550  K1-18- none vs. moderate = .9743  K1-18- none vs. severe = .1137 |
| Figure 4 | C | CAAR2:  n6dt- none vs. low = .7004  n6dt- none vs. med = .9523  n6dt- none vs. high = .3462  M22- none vs. low >.9999  M22- none vs. med = .9996  M22- none vs. high = .9991  K1-18- none vs. low >.9999  K1-18- none vs. med >.9999  K1-18- none vs. high = .9998  CAAR2.7:  n6dt- none vs. low = .9998  n6dt- none vs. med = .3544  n6dt- none vs. high = .1554  M22- none vs. low >.9999  M22- none vs. med >.9999  M22- none vs. high = .9998  K1-18- none vs. low >.9999  K1-18- none vs. med >.9999  K1-18- none vs. high >.9999 |
|  | D | CAAR2:  n6dt- none vs. low = .9927  n6dt- none vs. med = .2450  n6dt- none vs. high = .9997  M22- none vs. low = .9587  M22- none vs. med = .9915  M22- none vs. high = .1426  K1-18- none vs. low = .6021  K1-18- none vs. med >.9999  K1-18- none vs. high = .0554  CAAR2.7:  n6dt- none vs. low = .6106  n6dt- none vs. med = .3600  n6dt- none vs. high = .6931  M22- none vs. low = .9949  M22- none vs. med = .9859  M22- none vs. high = .5494  K1-18- none vs. low = .9021  K1-18- none vs. med = .5142  K1-18- none vs. high = .8041 |
|  | E | CAAR2:  IFNy:  n6dt- none vs. low >.9999  n6dt- none vs. med >.9999  n6dt- none vs. high >.9999  M22- none vs. low = .9917  M22- none vs. med = .9594  M22- none vs. high = .9855  K1-18- none vs. low >.9999  K1-18- none vs. med = .9990  K1-18- none vs. high = .9050  IL-2:  n6dt- none vs. low >.9999  n6dt- none vs. med = .9987  n6dt- none vs. high = .9984  M22- none vs. low = .9999  M22- none vs. med = .8322  M22- none vs. high = .7744  K1-18- none vs. low = .9870  K1-18- none vs. med = .8748  K1-18- none vs. high = .9932  IL-6:  n6dt- none vs. low = .9608  n6dt- none vs. med = .9998  n6dt- none vs. high = .9995  M22- none vs. low = .9963  M22- none vs. med = .9991  M22- none vs. high = .5868  K1-18- none vs. low = .1179  K1-18- none vs. med = .4231  K1-18- none vs. high = .1719  TNF:  n6dt- none vs. low >.9999  n6dt- none vs. med >.9999  n6dt- none vs. high >.9999  M22- none vs. low = .5346  M22- none vs. med = .5666  M22- none vs. high = .2772  K1-18- none vs. low = .9196  K1-18- none vs. med = .1413  K1-18- none vs. high = .9882  CAAR2.7:  IFNy:  n6dt- none vs. low = .9988  n6dt- none vs. med = .9985  n6dt- none vs. high = .9984  M22- none vs. low = .5146  M22- none vs. med = .1846  M22- none vs. high = .0152  K1-18- none vs. low = .6139  K1-18- none vs. med = .1266  K1-18- none vs. high = .0819  IL-2:  n6dt- none vs. low = .9925  n6dt- none vs. med = .9662  n6dt- none vs. high = .9674  M22- none vs. low = .5217  M22- none vs. med = .4114  M22- none vs. high = .1619  K1-18- none vs. low = .9964  K1-18- none vs. med = .9666  K1-18- none vs. high = .9408  IL-6:  n6dt- none vs. low = .9942  n6dt- none vs. med = .9784  n6dt- none vs. high = .9947  M22- none vs. low = .9731  M22- none vs. med = .2833  M22- none vs. high = .2551  K1-18- none vs. low = .9778  K1-18- none vs. med = .4642  K1-18- none vs. high = .3582  TNF:  n6dt- none vs. low >.9999  n6dt- none vs. med = .9999  n6dt- none vs. high = .9991  M22- none vs. low = .9996  M22- none vs. med = .9290  M22- none vs. high <.0001  K1-18- none vs. low = .9056  K1-18- none vs. med = .1671  K1-18- none vs. high = .6633 |
| Figure 5 | A | CAAR2:  n6dt- healthy vs. GD plasma 1 = .9358  n6dt- healthy vs. GD plasma 2 = .9963  n6dt- healthy vs. GD plasma 3 = .9560  M22- healthy vs. GD plasma 1 = .2597  M22- healthy vs. GD plasma 2 = .1267  M22- healthy vs. GD plasma 3 = .4520  K1-18- healthy vs. GD plasma 1 = .7011  K1-18- healthy vs. GD plasma 2 = .9551  K1-18- healthy vs. GD plasma 3 = .9664  CAAR2.7:  n6dt- healthy vs. GD plasma 1 = .9800  n6dt- healthy vs. GD plasma 2 = .9652  n6dt- healthy vs. GD plasma 3 = .9982  M22- healthy vs. GD plasma 1 <.0001  M22- healthy vs. GD plasma 2 <.0001  M22- healthy vs. GD plasma 3 = .0001  K1-18- healthy vs. GD plasma 1 <.0001  K1-18- healthy vs. GD plasma 2 = .0107  K1-18- healthy vs. GD plasma 3 = .4187 |
|  | B | CAAR2:  n6dt- healthy vs. GD plasma 1 = .8371  n6dt- healthy vs. GD plasma 2 = .8450  n6dt- healthy vs. GD plasma 3 = .9595  M22- healthy vs. GD plasma 1 = .9460  M22- healthy vs. GD plasma 2 = .9998  M22- healthy vs. GD plasma 3 = .9992  K1-18- healthy vs. GD plasma 1 = .7979  K1-18- healthy vs. GD plasma 2 = .9858  K1-18- healthy vs. GD plasma 3 = .9843  CAAR2.7:  n6dt- healthy vs. GD plasma 1 = .7201  n6dt- healthy vs. GD plasma 2 = .8784  n6dt- healthy vs. GD plasma 3 = .8590  M22- healthy vs. GD plasma 1 = .9539  M22- healthy vs. GD plasma 2 = .9998  M22- healthy vs. GD plasma 3 = .9999  K1-18- healthy vs. GD plasma 1 = .8813  K1-18- healthy vs. GD plasma 2 = .9968  K1-18- healthy vs. GD plasma 3 = .9889 |
|  | C | CAAR2:  IFNy:  n6dt- healthy vs. GD plasma 1 = .6717  n6dt- healthy vs. GD plasma 2 = .8343  n6dt- healthy vs. GD plasma 3 = .9903  M22- healthy vs. GD plasma 1 = .1184  M22- healthy vs. GD plasma 2 = .2680  M22- healthy vs. GD plasma 3 = .8858  K1-18- healthy vs. GD plasma 1 = .1623  K1-18- healthy vs. GD plasma 2 = .8884  K1-18- healthy vs. GD plasma 3 = .9578  IL-2:  n6dt- healthy vs. GD plasma 1 >.9999  n6dt- healthy vs. GD plasma 2 = .9963  n6dt- healthy vs. GD plasma 3 >.9999  M22- healthy vs. GD plasma 1 <.0001  M22- healthy vs. GD plasma 2 = .0054  M22- healthy vs. GD plasma 3 = .0014  K1-18- healthy vs. GD plasma 1 <.0001  K1-18- healthy vs. GD plasma 2 <.0001  K1-18- healthy vs. GD plasma 3 <.0001  IL-6:  n6dt- healthy vs. GD plasma 1 = .9437  n6dt- healthy vs. GD plasma 2 = .9684  n6dt- healthy vs. GD plasma 3 = .9433  M22- healthy vs. GD plasma 1 = .3388  M22- healthy vs. GD plasma 2 = .9995  M22- healthy vs. GD plasma 3 = .9334  K1-18- healthy vs. GD plasma 1 = .5033  K1-18- healthy vs. GD plasma 2 = .8288  K1-18- healthy vs. GD plasma 3 = .9886  TNF:  n6dt- healthy vs. GD plasma 1 = .9848  n6dt- healthy vs. GD plasma 2 = .9837  n6dt- healthy vs. GD plasma 3 = .9959  M22- healthy vs. GD plasma 1 = .0007  M22- healthy vs. GD plasma 2 = .0077  M22- healthy vs. GD plasma 3 = .9984  K1-18- healthy vs. GD plasma 1 <.0001  K1-18- healthy vs. GD plasma 2 <.0001  K1-18- healthy vs. GD plasma 3 <.0001  CAAR2.7:  IFNy:  n6dt- healthy vs. GD plasma 1 = .8114  n6dt- healthy vs. GD plasma 2 = .9491  n6dt- healthy vs. GD plasma 3 >.9999  M22- healthy vs. GD plasma 1 = .0923  M22- healthy vs. GD plasma 2 = .0811  M22- healthy vs. GD plasma 3 = .4182  K1-18- healthy vs. GD plasma 1 = .0206  K1-18- healthy vs. GD plasma 2 = .0140  K1-18- healthy vs. GD plasma 3 = .0397  IL-2:  n6dt- healthy vs. GD plasma 1 = .9961  n6dt- healthy vs. GD plasma 2 = .9953  n6dt- healthy vs. GD plasma 3 = .9962  M22- healthy vs. GD plasma 1 = .0003  M22- healthy vs. GD plasma 2 = .0004  M22- healthy vs. GD plasma 3 = .0002  K1-18- healthy vs. GD plasma 1 <.0001  K1-18- healthy vs. GD plasma 2 <.0001  K1-18- healthy vs. GD plasma 3 <.0001  IL-6:  n6dt- healthy vs. GD plasma 1 = .9485  n6dt- healthy vs. GD plasma 2 = .9999  n6dt- healthy vs. GD plasma 3 = .9274  M22- healthy vs. GD plasma 1 = .5123  M22- healthy vs. GD plasma 2 = .9649  M22- healthy vs. GD plasma 3 = .9766  K1-18- healthy vs. GD plasma 1 = .7519  K1-18- healthy vs. GD plasma 2 >.9999  K1-18- healthy vs. GD plasma 3 = .7024  TNF:  n6dt- healthy vs. GD plasma 1 = .8115  n6dt- healthy vs. GD plasma 2 = .7465  n6dt- healthy vs. GD plasma 3 = .9329  M22- healthy vs. GD plasma 1 = .0013  M22- healthy vs. GD plasma 2 = .0007  M22- healthy vs. GD plasma 3 = .3823  K1-18- healthy vs. GD plasma 1 <.0001  K1-18- healthy vs. GD plasma 2 <.0001  K1-18- healthy vs. GD plasma 3 <.0001 |
| Supplemental Figure | C | nalm6 vs. M22 <.0001  nalm6 vs. K1-18 = .0044 |

**Supplementary Table 1.** Exact statistical values for data in the manuscript.
